# Strategies for Establishing Policy, Environmental, and Systems-Level Interventions for Managing High Blood Pressure and High Cholesterol in Health Care Settings: A Qualitative Case Study

**Published:** 2008-06-15

**Authors:** Dyann Matson Koffman, Sharon A Granade, Victoria V Anwuri

**Affiliations:** Centers for Disease Control and Prevention, National Center for Chronic Disease Prevention and Health Promotion; Centers for Disease Control and Prevention, National Center for Preparedness, Detection, and Control of Infectious Diseases, Atlanta, Georgia; Robert Wood Johnson Foundation Diabetes Initiative National Program Office, St. Louis, Missouri. At the time of this study, Ms Anwuri was affiliated with the Association of Schools of Public Health and Centers for Disease Control and Prevention, National Center for Chronic Disease Prevention and Health Promotion, Atlanta, Georgia

## Abstract

**Introduction:**

Policy, environmental, and systems-level interventions are part of a comprehensive approach to managing high blood pressure and high cholesterol, which are key risk factors for heart disease and stroke. In this qualitative case study, we identified clinical practices in health care organizations that used policy, environmental, or systems-level interventions to improve patient outcomes for these conditions. Our 4 objectives were to describe 1) policy, environmental, and systems-level interventions; 2) enabling factors and barriers that affected implementation; 3) methods for evaluating the success of the intervention; and 4) lessons learned from the health care practices that implemented these interventions.

**Methods:**

Through literature review and expert guidance, we identified 34 health care practices that used policy, environmental, and systems-level interventions to manage high blood pressure and high cholesterol. In 2003, we conducted case study interviews with key informants for 9 health care practices that 1) demonstrated improved patient outcomes for blood pressure or cholesterol; 2) implemented the interventions for at least 1 year; and 3) remained committed to sustaining or institutionalizing interventions. We taped and transcribed the interviews and used Centers for Disease Control and Prevention EZ-Text software (www.cdc.gov/hiv/software/ez-text.htm) to code, categorize, and analyze the responses.

**Results:**

The health care practices we studied implemented specialized lipid clinics, disease management programs, physician reminder systems, and participation in the Health Resources and Services Administration's Bureau of Primary Care Health Disparities Collaboratives. All practices used comprehensive systems for patient care that were well-defined, measurable, and linked to desirable patient outcomes. Most relied on data systems to identify patients targeted for the interventions and practice areas that needed improvement, and to track the progress of patients and practitioners in meeting goals. Factors contributing to success included support for patient self-management, interventions integrated into the practice's daily work flow to make implementation easier for staff, leadership and staff commitment, and community involvement.

**Conclusion:**

Comprehensive policy, environmental, and systems-level interventions for patient care can be effective in controlling chronic conditions such as high blood pressure and high cholesterol.

## Introduction

Coronary heart disease and stroke are the first and third leading causes of death in the United States (1) and major causes of disability (1,2). The American Heart Association and the American Stroke Association estimate that the total direct and indirect cost of cardiovascular disease (CVD) in the United States in 2007 was $438 billion (2). Much of the burden of heart disease and stroke could be eliminated by preventing or reducing 7 major risk factors — high blood pressure, high cholesterol, obesity, tobacco use, diabetes, physical inactivity, and poor diet (3,4). For example, a reduction in systolic blood pressure of 12 to 13 mm Hg over 4 years of follow-up can reduce heart attacks by 21%, strokes by 37%, and all deaths from coronary vascular disease by 25% (5). Furthermore, a 10% decrease in total cholesterol may reduce the incidence of coronary heart disease by as much as 30% (6,7). However, only an estimated 31% of adults with high blood pressure (8) and 18% of those with high cholesterol (9) have these conditions under control. The 2004 estimated prevalence of high blood pressure at or above 140/90 mm Hg in U.S. adults aged 20 and older was 33.6%, and total cholesterol at or above 200 mg/dL was 48.4% (2). The *Healthy People 2010* targets are to reduce the proportion of adults with high blood pressure to 16% and to lower the proportion of adults with elevated total cholesterol to 17% (10).

Why is it such a challenge to prevent and control high blood pressure and high cholesterol? The literature cites many reasons (11-15). Some articles focus on patient-related factors, such as noncompliance with treatment regimens because of medication side effects, complexity of the drug regimen(s), and lack of awareness of the need for long-term therapy (11-13). Others focus on the failure of physicians to consistently comply with evidence-based guidelines (14). According to Wagner, Austin, and Von Korff, clinical practices are not structured to adequately care for patients with chronic conditions (15):

Medical practices, especially those in primary care, are generally organized to respond to the acute and urgent needs of their patients, or symptom-relieving treatments. . . . This leaves little time or intellectual energy for addressing the less urgent, but nevertheless predictable, needs of patients with chronic illness in managing their conditions and preventing deleterious sequelae.

Policy, environmental, and systems-level (PES) interventions constitute a paradigm shift in clinical care from earlier health care models and represent an ecological model that links PES changes with behavior changes at the individual level to prevent heart disease and stroke (16-18). Policy interventions include laws, regulations, and formal and informal rules adopted within specific organizations (19). For example, health care organizations can have treatment policies to implement evidence-based national clinical guidelines such as those of the Seventh Report of the Joint National Committee on Prevention, Detection, Evaluation, and Treatment of High Blood Pressure (JNC 7) (20); the Third Report of the Expert Panel on Detection, Evaluation, and Treatment of High Blood Cholesterol in Adults (ATP III Final Report) (21); and the 2002 update to the American Heart Association (AHA) guidelines (22) and the 2001 update to the  AHA/American College of Cardiology guidelines (23).

Environmental interventions are changes to the economic, social, or physical environments (19). Examples include making community resources available and known to the public and creating an environment that allows people to make healthier choices.

Systems-level interventions are changes affecting the way an organizational system operates. Within the health care setting, these interventions generally focus on changing the ways in which health services are delivered and may include delegating responsibility for key care functions to nonphysician members of the health care practice, putting systems in place to ensure appropriate follow-up with patients, and providing regular feedback to physicians on how well they manage their patients' conditions (24).

A 1996 literature review (15) examined comprehensive approaches to reorganizing the delivery of care to improve the outcomes of patients with chronic illness. The review’s authors noted that successful interventions shared the following elements: 1) explicit protocols based on evidence-based guidelines; 2) practice organization to meet the needs of patients who require more time, a broad array of resources, and closer follow-up; 3) systematic attention to providing patients with information for behavioral change; 4) ready access to necessary expertise; and 5) supportive information systems (15). These elements, which also could be considered PES interventions, are reflected in Edward H. Wagner’s Chronic Care Model (25). This model for improving the care of patients with chronic disease has essential elements grouped in 6 areas: 1) the community (resources and policies), 2) the health care system (organization of health care), 3) support for patient self-management, 4) design for delivering health services to patients, 5) support for physician decisions, and 6) clinical information systems.

In a 1997 review of interventions to prevent cardiopulmonary disorders among patients in health care settings, Ockene et al (26) concluded that traditional education and training programs for health care providers combined with reminders systems, feedback to providers, and practice guidelines are most effective for delivering preventive interventions. They also found that the effectiveness of providers is optimized when the intervention uses a team of both nonphysicians and physicians to counsel patients on prevention and when providers support the control of risk factors through multiple patient visits, follow-up telephone calls to the patient, or referrals to specialists as appropriate.

We have identified other promising quasi-experimental or experimental studies published from 1997 through 2007 describing interventions in health care settings that contribute to preventing and controlling high blood pressure, high cholesterol, heart disease, and stroke (14,20-23,27-72). Elements of interventions that showed improvement in outcomes for high blood pressure or high cholesterol included 1) providers that adhere to standardized protocols that are consistent with evidence-based national guidelines (20-23,28-44); 2) multidisciplinary clinical care teams that deliver elements of quality patient care, such as routine and consistent screening, assessment, counseling, and patient follow-up contacts, to control risk factors (32,33,38,45-48); 3) treatment and prevention clinics that deliver focused management of care (31,33-37,49-54); 4) electronic medical record systems, automatic prescription systems, and paper and electronic reminder systems for health care providers (14,55-63); and 5) patient education (61,62,65-71). National treatment guidelines recommend that clinical prevention and treatment services include these 5 elements (54,55,58,59,63,64,68,71).

### Purpose of study

The purpose of our case study was to identify and describe promising health care practices (PHPs) and the PES interventions these practices used to control high blood pressure and high cholesterol. We defined a PHP as one that had practice-based data showing positive outcomes from interventions to reduce risk factors related to heart disease and stroke but whose data may not yet have been tested in controlled studies to allow for generalizable results (16). We also identified the strategies that led to successful adoption and implementation of these PES interventions, the methods used to evaluate progress, and lessons learned.

## Methods

We searched peer-reviewed literature databases (ABI/Inform, Ann Arbor, Michigan; Lexis/Nexis, New York, New York; OVID, New York, New York; and the National Library of Medicine’s PubMed, Bethesda, Maryland) for articles published from 1992 through 2003 to identify studies of PES interventions in health care settings that were focused on the prevention of heart disease and stroke. We also used Google (www.google.com) to identify practices implementing PES interventions that had received special recognition, such as the C. Everett Koop award for exemplary health care programs. In addition, we selected a variety of experts from among participants in national conferences and our own professional contacts, including staff from state health departments, representatives from business groups and health associations, and researchers, to identify PHPs that were using PES interventions. The search generated 644 studies of PHPs that used PES interventions. We narrowed the list to 34 studies that reported all 3 of the following: 1) data on improved patient outcomes in the control of blood pressure (<140/90 mm Hg) and cholesterol (<200 mg/dL for total cholesterol or <100 mg/dL for low-density lipoprotein cholesterol [LDL]), 2) incorporation of the interventions for at least 1 year, and 3) organizational commitment to sustaining or institutionalizing the interventions. We made initial phone calls to gather information if it was not publicly available and then used the following criteria to rank and select 9 of the 34 PHPs: 1) use of a control or comparison group in the study, 2) outcomes that were statistically or clinically significant, 3) incorporation of national JNC 7 (20) and ATP III (21) guidelines, and 4) study populations that were at high risk for heart disease and stroke. Using in-depth telephone interviews, we conducted a case study with key informants (physicians, other health-care practitioners, and administrators) of the 9 PHPs selected in order to identify and learn about the factors and strategies that worked for establishing the PES interventions (73). The Centers for Disease Control and Prevention (CDC) taped and transcribed the interviews and used CDC EZ-Text software (provided free at www.cdc.gov/hiv/software/ez-text.htm) to code, categorize, and analyze responses to the interviews.

The 9 PHPs were the Laurel Health System's CVD Collaborative (Wellsboro, Pennsylvania); Health Management Corporation (Richmond, Virginia) for Blue Cross Blue Shield (BCBS) of Delaware (Wilmington, Delaware); Kaiser Permanente (Cleveland, Ohio); Mayo Clinic's Division of Community Internal Medicine (Rochester, Minnesota); Robeson Healthcare Corporation's CVD Collaborative (Lumberton, North Carolina); Midwest Heart Specialists (Chicago and Rockford, Illinois); MVP Health Care (Schenectady, New York, and Williston, Vermont); Drug Therapy Management, Inc (Pharmacist-Managed Lipid Clinic, Greensboro, North Carolina); and Partners for Better Health of Health Partners (Minneapolis, Minnesota).

## Results

The Table summarizes the PES interventions implemented in each of the 9 PHPs included in our study, and we describe the study design and reported patient outcomes in the Appendix. Four of the practices also reported their outcome data for high blood pressure and high cholesterol control in 5 studies in peer-reviewed journals (32,33,54,55,64).

Key informants described the interventions in their PHPs, including 1) factors leading to their implementation, 2) models used in their design, 3) patient outcomes, 4) strategies for measuring progress, 5) costs, and 6) lessons learned.

### Factors leading to interventions

The primary reasons PHPs gave for implementing the interventions were the realization and concern that patients' lipid concentrations (especially LDL cholesterol) or blood pressures were not adequately controlled, as judged by using national guidelines (e.g., JNC 7 [20], ATP III [21]), and that the compliance of practice providers with these guidelines was inconsistent. This realization often stemmed from reviewing patient charts and evaluating the way health care was delivered within the informants' organizations. Key informants reported that some practitioners admitted that before they analyzed their patient charts, they had believed they were adequately following guidelines to help patients manage their conditions and were surprised to find they were not.

Some key informants said the frequent diagnoses of heart disease within their patient population and the related costly procedures led their organization to implement PES interventions. Other informants said they were influenced by data in the literature reporting on the U.S. burden of heart disease and the positive outcomes from PES interventions, particularly for reducing risk factors such as high cholesterol and high blood pressure. Informants from 2 PHPs mentioned that their decision to implement interventions was influenced by changes in the health care commercial market, particularly the move toward managed care, combined with their need to stay competitive. Practitioners in some PHPs introduced interventions that would help them more efficiently manage CVD in patients without adversely affecting the time spent on other responsibilities. Communities were the impetus behind some of the interventions. For example, a community group in Pennsylvania identified diabetes and CVD as major health problems in its community; because of the group's commitment to reducing the impact of these diseases in the community, it worked with the Laurel Health System to spearhead and move its diabetes and cardiovascular collaboratives forward.

### Descriptions of the interventions

Key informants described a wide array of PES interventions that had been implemented in their organizations, all of which were based on nationally recognized guidelines and best practices. The primary types of interventions implemented were automated physician reminder systems, specialized lipid clinics, collaboratives, and disease management programs (described below). The goals of the interventions centered on providing physicians with the tools to adequately treat patients with high blood pressure or high cholesterol, empowering patients with the skills to manage their conditions, achieving the goals referenced in the national guidelines, and, ultimately, preventing complications related to CVD.

#### Automated physician reminder systems integrated into electronic medical records

Kaiser Permanente, Ohio, integrated physician reminders into its electronic medical records system to support physician decision making at the time of each patient visit. This made it easier for physicians to access patient data during the visit, to be alerted when a patient's cholesterol or blood pressure was uncontrolled, to determine when testing for lipids or other purposes should be performed, and to adjust medications as needed.

#### Specialized lipid clinics

Two PHPs used specialized lipid clinics to manage their patients' cholesterol and triglyceride values. Lipid clinics provide patient-focused care to educate patients and to assist them in setting self-management goals for changes in lifestyle. In addition, these clinics track patient status as measured by cholesterol values, medications prescribed, patient compliance with medication regimens, follow-up visits, and other factors that determine whether comprehensive care is being offered. Some clinics also adjust medications based on evidence-based algorithms and the approval of the physician. Midwest Heart Specialists established a physician-directed lipid clinic that nurses managed for some patients with high cholesterol. The results of this lipid clinic were so impressive (54) that the practice developed a virtual lipid clinic accessible to all providers that incorporated the process used in the nurse-managed clinic into an electronic record system with a cholesterol management tool (55). The development of the virtual lipid clinic enabled patients from throughout the practice to benefit from this intervention. Drug Therapy Management, Inc, a pharmacist-managed lipid clinic contracted with a cardiology clinic, also reported successful patient outcomes (33).

#### Collaboratives

A collaborative is an integrated and collaborative national effort to eliminate disparities and improve health care delivery systems. Collaboratives are designed to help health care organizations improve the care of their patients with CVD and other chronic diseases. Collaboratives promote evidence-based health care strategies for quality improvement based on the Chronic Care Model and on the Plan-Do-Study-Act (PDSA) cycles described below (74). They also emphasize sharing experiences among participating organizations to enable them to learn from one another. Three PHPs participated in a collaborative. Mayo Clinic's Division of Community Internal Medicine in Minnesota participated in a collaborative sponsored by the Institute for Clinical Systems Improvement (ICSI) (www.icsi.org/). As part of this collaborative, this PHP implemented the ICSI Guidelines (75) for blood pressure control, which are based on JNC-VI Guidelines (76), and redesigned the system by which nurses and medical assistants screened and managed patient blood pressure. Robeson Healthcare Corporation and Laurel Health System implemented the Prevention of Diabetic and Cardiovascular Disease Collaborative of the Health Resources and Services Administration's (HRSA's) Bureau of Primary Care Health Disparities Collaboratives (www.healthdisparities.net/hdc/html/collaborativesOverview.aspx).

#### Disease management programs

Three PHPs (Partners for Better Health of Health Partners, MVP Health Care, Health Management Corporation/BCBS of Delaware) described their disease management programs for proactive, comprehensive management of their patients with CVD. Disease management programs, which are disease-specific, provide care by using such strategies as population identification processes, evidence-based practice guidelines, collaborative practice models that include both physicians and providers of support services, patient self-management and education, measurements of process and outcomes, evaluation and management, and routine reporting and feedback loops (77).

### Health models used for designing interventions

Four of the 9 PHPs incorporated the Chronic Care Model along with PDSA cycles for quality improvement when designing their PES interventions. PDSA cycles rely on simple measurements to monitor the effect of small changes over time, which then can build into larger improvements through successive quick cycles of change. Although informants for the other 5 PHPs did not explicitly mention using the Chronic Care Model, their interventions often contained some of this model's elements, such as use of protocols based on evidence-based guidelines, reorganization of health care delivery, use of clinical information systems, and support for patient self-management.

Two PHPs (Partners for Better Health of Health Partners and Health Management Corporation/BCBS of Delaware) modeled their patient support components according to where patients were on the health continuum:

One model that we've used is a model of the health continuum [that] describes what we're trying to do with health improvement, and it goes from keeping people from advancing into stages of disease. So we look at our programs from primary prevention all the way through the care of the [patient] population with the highest risk and mortality. (informant, Partners for Better Health of Health Partners)

Such a model is patient-focused and approaches health promotion and prevention of risk factors at all stages of disease. Along these lines, some PHPs used nurses to regularly interact with patients via telephone or other avenues to offer education and support.

Some interventions were modeled after existing programs that had proven successful. For example, MVP Health Care implemented a CVD management program patterned after other programs already in place for its patients with asthma and diabetes.

### Patient outcome data

Patient outcome data for these PES interventions are limited to responses from the interviews with key informants (see Appendix) and the 5 studies reported in peer-reviewed journals (32,33,54,55,64). Informants from all PHPs reported that their interventions were very successful at increasing providers' compliance with guidelines and in increasing the percentage of patients who met their cholesterol or blood pressure goals, complied with medication regimens, or set and achieved goals for self-management. Informants also reported improvements in the delivery of health care, which led to an increase in the consistency and quality of patient care and more productive use of providers' time. Some informants reported that these improvements decreased utilization of various forms of health care, such as inpatient admissions and emergency room visits, and resulted in significant cost savings to health care plans.

### Measuring progress

Informants from the PHPs noted that they measured varying combinations of outcomes and processes for quality improvement. The indicators that the programs used to measure the various PES interventions are described below. Many informants stated that the organization must first agree on its definition of success:

Critical in our start was that we had to come to a common definition of success . . . not only [to] define success, but also [to] measure success. And that was coming to agreement on the different . . . outcomes measures, process measures, and end results that would define our success for everyone. So that we were all . . . going after the same goals. (Informant, Health Management Corporation/BCBS of Delaware)

#### Outcomes measures

Among the patient outcome measures PHPs reported were monitoring control of lipids, glucose, and blood pressure among patients at risk and patients achieving self-management goals, such as weight loss, smoking cessation, and participation in regular exercise. Some PHPs also evaluated use of aspirin, angiotensin-converting enzyme inhibitors, and beta-blockers in patients with heart disease. Many PHPs also measured utilization rates for health care, such as emergency room visits, hospitalizations, and the pharmacy. In addition, Partners for Better Health of Health Partners monitored the percentage of patients who had all modifiable risk factors managed optimally. Drug Therapy Management, Inc, monitored the number of recurrent cardiovascular events, and Midwest Heart Specialists calculated the number of cardiovascular events prevented or lives saved.

#### Process measures

Process measures included measuring physicians' compliance with policies and guidelines, patients' compliance with medications and plans of care, and the percentage of patients screened for abnormal lipids, blood pressure, glucose, and glycosylated hemoglobin. PHPs that incorporated PDSA cycles conducted mini-studies to evaluate the effectiveness of each step of the intervention. For example, as part of redesigning the way it delivered patient care, Mayo Clinic delegated responsibility to licensed practical nurses (LPNs) and medical assistants (MAs) for taking patients' blood pressure and referring patients with elevated values for a follow-up visit with a registered nurse. Before delegating this responsibility to the LPNs and MAs, the clinic first monitored the number of times the LPN or MA successfully recognized high blood pressure and made the referral.

#### Choosing measures

Informants for some PHPs explained how they chose measures and why they chose to evaluate certain ones. They chose measures they believed would help them improve the quality of care for patients, decrease expenses, or have the most impact on health outcomes. Often they chose to evaluate measures to help them comply with standards set by organizations such as the National Committee for Quality Assurance, HRSA's Bureau of Primary Health Care, or the National Cholesterol Education Program:

We felt that we could make the most impact on the lipid profile, so we chose the lipid one [as an optional parameter to measure]. (informant, Robeson Healthcare Corporation)

#### Information systems

Most PHPs used information technology systems to continuously monitor selected measures and to provide regular feedback to health care practitioners, organization management, or HRSA's Bureau of Primary Health Care (Health Disparities Collaboratives). The feedback showed areas in need of improvement and indicated whether goals were achieved. The PHPs subsequently used the feedback to make improvements in various components of the intervention. One PHP informant described the use of statistical process control (SPC) to evaluate which processes needed improvement:

Using SPC, you could tell if some of the measures were basically more than 2 standard deviations from the desired goal. In which case, in SPC terminology, that means you need to change your processes. (informant, Kaiser Permanente, Ohio)

#### Barriers to measurements

PHPs encountered some difficulties in measuring progress or success of interventions. Among these were a lack of resources for gathering necessary data, goals that proved too ambitious, and variation in physician behavior. Informants said they sometimes had problems with their computer systems. Some said that entering new patient data skewed all previously entered measurement data, making it difficult to evaluate improvements over time. They also said the lack of standardized codes for laboratory tests or other procedures sometimes made it difficult to merge data from different sources.

### Costs of interventions

When asked to estimate the financial costs of their PES interventions, PHP informants sometimes had difficulty assigning a total monetary value to the interventions. This was because many components of the interventions were phased in over a period of time, and others were incorporated into regular staff duties:

It's really hard to answer that because it [the intervention] becomes part of everybody's work and it becomes embedded into the activities across the entire spectrum of the organization. (informant, Partners for Better Health of Health Partners)

The costs of interventions included personnel time and resources devoted to planning and goal setting, implementation of the interventions, and ongoing activities to sustain the interventions. Some interventions involved significant time and effort for many people throughout the organization, often incurring substantial expenditures as measured by decreased time to devote to other services.

One informant described the many variables that could affect the cost of setting up a lipid clinic:

The [lipid] clinic costs are dependent on how they [are] set up. . . . The cost of putting in a system depends on what you're doing. If you're putting in a paper system, it costs almost nothing. If you put in a computer system, it can cost several thousand dollars per physician. (informant, Midwest Heart Specialists)

Some PHPs attempted to estimate the cost for certain components of their interventions. For example, the costs ranged from $10,000 to $15,000 for a pharmacy-managed lipid clinic to $700,000 for an automated medical records system with physician reminders for a patient population of approximately 300,000. Robeson Healthcare Corporation expressed the opinion that interventions were worth the cost if they ultimately prevented heart attacks, strokes, and intensive hospitalizations. Midwest Heart Specialists developed a virtual computer-based lipid clinic to help reduce costs associated with its more intensive nurse-managed lipid clinic.

Some informants said that the PES interventions, once implemented, actually increased productivity and freed up time for physicians and other staff. For some interventions, the costs were offset by help from other resources. For example, some PHPs received funds through special funding grants for intervention start-up costs. Health insurance companies enabled other interventions by reimbursing intervention services or by allowing their members to have access to intervention programs. Because of their proven success with interventions, some PHPs have been able to negotiate better health insurance reimbursement rates for intervention-related services.

### Lessons learned and recommendations

Key informants from the PHPs offered numerous recommendations to those interested in implementing similar interventions.

**Design and implementation of interventions**

- Generate awareness of the intervention among the staff, community (when applicable), and other stakeholders.
- Assure that all the stakeholders' visions for the intervention program are in alignment.
- Plan and organize details of the implementation, including resources that will be needed.
- Develop a system for the performance of work processes and integrate these processes into the work flow for delivering health services. Coordinate the operations among all the departments in the organization that will be involved with the interventions.
- Start small and persist; do not let desire for perfection be a stumbling block.
- Plan to sustain the interventions, which should include ongoing training for the staff.

**Staffing**

- Get the right people on the health care team:

Pick people [who] are interested and enthusiastic to start with; don't go for your toughest nuts first. (informant, Mayo Clinic)

- Interact with staff and other key players to communicate how the protocols will work.
- Listen to and understand the needs of staff involved in implementing the interventions.
- Delegate work responsibilities to members of the health care team:

Get everyone on the team working at their maximum level. (informant, Mayo Clinic)

**Patient focus**

- Provide patient education with a focus on changing behaviors:

It's really working with people where they are and moving them along the continuum from inaction to actually taking action. (informant, Health Management Corporation/BCBS)

- Foster relationships with patients:

[B]ecause it doesn't matter if you're really intelligent; if you can't communicate well with the patient and be congenial, friendly, and likable, then it's not going to work well. The patient is not going to follow what you say. I think that's what has helped patients be compliant, is having someone that's very caring and really concerned about them. (informant, Drug Therapy Management, Inc)

## Discussion

On the basis of these case studies and reported outcome data, we concluded that practices that use comprehensive systems of patient care can be effective in controlling high blood pressure and high cholesterol. The PES interventions included 1) automated physician reminder systems, 2) specialized lipid clinics, 3) collaboratives, and 4) disease management programs. The common thread for these interventions is that they all incorporated comprehensive systems for patient care with processes that were well-defined, measurable, and linked to desirable patient outcomes. Most relied on data systems to identify patients targeted for the interventions and areas in need of improvement and to track progress. Most of them also included elements of the Chronic Care Model, and some said they used PDSA cycles to measure the effectiveness of each step of change.

Factors critical to success of interventions included support for patient self-management and education, interventions integrated easily into the daily work flow to make it easier for staff to implement them, and leadership and staff commitment. Other important factors included involving staff throughout the organization and community groups in the planning and implementation stages, effective communication with health care providers and other staff, having ongoing training of staff, and keeping the intervention project high on the agenda so that motivation for sustaining it would not be lost. Many of the key informants also noted the importance of enlisting the right people to be on the intervention team.

These PES interventions may not be applicable to all health care settings. Indeed, we note that the 9 PHPs we studied were often affiliated with larger health care plans that could afford these sometimes costly interventions. Smaller health care practices may not have the necessary resources.

### Limitations and strengths of the case method

Although most of the PHPs studied relied on noncontrolled evaluations, 4 of them used experimental or quasi-experimental designs to evaluate the PES interventions. Although all of the information and data for this article were gathered through an interview process, 4 of the practices also reported their outcome data for high blood pressure and high cholesterol control in 5 studies in the peer-reviewed literature (32,33,54,55,64). We identified common factors among the PHPs that contributed to their success in implementing the interventions, but this information was based on only 9 practices. Furthermore, our case method did not allow us to determine which PES interventions or combinations of interventions were most effective. We recommend a meta-analysis of PES interventions in the literature.

### Recommendations

Further research is needed to evaluate the effectiveness of various PES interventions and to determine how smaller health care practices can adopt them. In addition, research is needed to determine the actual costs of putting these systems in place and their impact on improving health conditions. Many informants were not aware of the total costs of implementation. Health care executives may be hesitant to initiate similar interventions in their practices if the return on investment is uncertain. Studies are also needed to determine whether PES interventions can sustain positive patient outcomes, avert or delay heart disease or stroke, and improve patients' health, quality of life, and productivity.

### Conclusion

The 9 PHPs we studied that used and institutionalized PES interventions for at least a year reported positive outcomes for controlling high blood pressure and high cholesterol. We are among the first to use case studies to synthesize the practical experiences, lessons learned, and recommendations of PHPs using a variety of PES interventions. These results verify the value of PES interventions reported in the literature, and they have important implications for clinicians and for policy makers. The results show clinicians that certain strategies and factors are critical for establishing comprehensive systems of care, which can lead to positive patient outcomes. Policy makers may want to consider initiatives that require health care practices to adopt these systems of care.

## Figures and Tables

**Table.** Policy, Environmental, and Systems-Level Interventions Implemented for Blood Pressure and Cholesterol Control Reported by Key Informants at 9 Health Care Practices, 2003^a^

**Intervention**	KP	DTM	MHS	Mayo	RHC	LHS	HP	HMC/BCBS	MVP
Disease management program							●	●	●
Participation in collaborative				●	●	●			
Specialized lipid clinics		●	●						
Automated physician reminders	●		●		●				
Electronic medical records	●		●					●	
National guidelines	●	●	●	●	●	●	●	●	●
Patient education and self-management goals		●	●	●	●	●	●	●	●
Nurse telephone lines			●			●	●	●	●
Treatment protocols or algorithms		●	●						
Patient tracking system or registry	●	●	●		●	●	●	●	●
Patient flow sheets		●	●	●	●				
Appointment reminders	●	●	●						●
Multidisciplinary team			●	●	●	●		●	
Progress reports or report cards			●		●	●	●		●
Chronic care model				●	●	●	●		
Plan-Do-Study-Act cycles				●	●		●		
Care delivery redesign			●	●	●				

KP indicates Kaiser Permanente, Cleveland, Ohio; DTM, Drug Therapy Management, Inc, Greensboro, North Carolina; MHS, Midwest Heart Specialists, Chicago and Rockford, Illinois; Mayo, Mayo Clinic, Division of Community Internal Medicine, Department of Medicine, Rochester, Minnesota; RHC, Robeson Healthcare Corporation, Lumberton, North Carolina; LHS, Laurel Health System, Wellsboro, Pennsylvania; HP, Health Partners Health Plan/Disease Management Program, Minneapolis, Minnesota; HMC/BCBS, Health Management Corporation, Richmond, Virginia/Blue Cross Blue Shield of Delaware, Wilmington, Delaware; MVP, MVP Health Care, Schenectady, New York and Williston, Vermont.

a Data were collected through telephone interviews with key informants (physicians, other health care practitioners, and administrators) at the 9 participating health care organizations.

## Appendix. Intervention Outcomes and Interview Comments, Case Study of 9 Health Care Practices, 2003

Outcomes of case study interviews with key informants for 9 health care practices were reported in studies published from 1992 through 2002 and identified through a literature review by the authors (32,33,54,55,64). Key informants at 9 health care organizations were interviewed in 2003. Following is a summary of these interviews arranged by intervention.

### Automated Physician Reminder Systems Integrated Into Electronic Medical Records

**Practice: Kaiser Permanente, Cleveland, Ohio**

**Outcomes reported:** Intervention group had statistically significant improvement in cholesterol outcomes (64). Study design was nonexperimental; repeat cross-sectional design-time series.

**Interview comment:**

"[T]here was better control of cholesterol levels in heart disease patients after we turned on [physician] reminders. . . . [At the end of 2001, there was] a 40% increase in cholesterol control. . . . [W]e've seen improvement in low-density lipoprotein (LDL) screening and control."

### Specialized Lipid Clinics

**Practice: Drug Therapy Management, Inc, Greensboro, North Carolina **

**Outcomes reported:** Intervention group had statistically and clinically significant improvement in cholesterol outcomes compared with control group (33). Study design was quasi-experimental.

**Interview comment:**

"[T]here are about 500 patients in the study. . . .[A]bout 93% [met] their LDL goal levels of less than 100 [mg/dL]. . . . So it's been very successful."

**Practice: Midwest Heart Specialists, Chicago and Rockford, Illinois **

**Nurse-Managed Lipid Clinic**

**Outcomes reported:** Intervention group had clinically significant improvement in cholesterol outcomes compared with control group (54). Study design was quasi-experimental.

**Interview comment:**

"Following the initial evaluation, 71% of patients in the lipid clinic were at LDL goal, versus 22% of patients at LDL goal in the rest of the cardiology practice, and 11% of patients at goal in general practices throughout the country (at that time)."

**Virtual Lipid Clinic**

**Outcomes reported:** Intervention group had clinically and statistically significant improvement in cholesterol outcomes compared with control group (55). Study design was experimental, with randomized selection of patients for intervention and control groups.

**Interview comments:**

"[L]ed to much higher quality patient care on a much more consistent basis practice-wide . . . very effective to a much larger group of patients."

"Approximately 60% of patients are at LDL goal throughout the practice, including nurse-managed and virtual lipid clinics."

### Participation in Collaboratives

**Practice: Mayo Clinic, Division of Community Internal Medicine, Department of Medicine, Rochester, Minnesota **

**Outcomes reported:** Intervention group had clinically and statistically significant improvement in outcomes for blood pressure (32). Study design was experimental.

**Interview comments:**

"We showed fairly consistently about a 5% difference in control rates between physicians using this model and physicians not using this model."

"[W]e've seen sort of a sustained improvement in our control rates at least for the 3 or so initial years of this study. I think we went somewhere from about a baseline of 30% to somewhere up in about the 45% range."

**Practice: Robeson Healthcare Corporation, Lumberton, North Carolina **

**Outcomes not published**

**Interview comments:**

"Some of the more promising results of the collaborative are improvement in the outcome measure of having blood pressure under control and improvements in self-management goal setting. Currently (2003), in our self-management goal setting for the cardiovascular registry . . . about 34% of our [population of focus] has had a self-management goal set. . . . [On] the spread population, 35% of 3000 people have had a self-management goal set. And that was something that 3 years ago we were not doing at all."

In a population of focus of 142 people over 3 years, "45% of those people have a blood pressure under 140/90. I think the national average is around 20%, and we have been as high as 57%, which is almost 3 times that national average. And so that outcome measure, we have sustained that."

"[I]f you look at our spread population, right now, we're at 32% of 3000 people . . . who have a blood pressure under control of 140/90. That number has been as high as 42%, but that was back when we had 1000 people in the registry. So we've steadily climbed from [about] 15% up to 32% over the last year while maintaining over 3000 people in that registry."

**Practice: Laurel Health System, Wellsboro, Pennsylvania **

**Outcomes not published**

**Interview comments:**

"[O]ne of our goals was [getting] patients' blood pressure under 140/90 mm Hg. The national goal is 50%. Right now we are at about 40% of our [population of focus] that have made goal."

"[O]ur latest data [show that] we have 431 patients in the cardiovascular registry in that health center and approximately 40% are at goal, whereas the spread [population] is 1783, and they are a little over 50% [at goal], so their total is close to 50%."

### Disease Management Programs

**Practice: Health Partners Health Plan, Minneapolis, Minnesota **

**Outcomes not published**

**Interview comment:**

"[W]e've seen a significant shift toward comprehensive care. . . . [A]re they getting their blood pressure in control and do they also have their lipid levels not only done, but in control, and we're actually measuring based on that whole picture and not just one component of that for a person. So we think best care equals having all of that done and so it has gone up in the last few years, but it certainly has a long way to go."

**Practice: Health Management Corporation, Richmond, Virginia, for Blue Cross Blue Shield of Delaware, Wilmington, Delaware **

**Outcomes not published**

**Interview comments:**

"[H]ealth status and health process measures like getting the testing and the results, we see over time improvements in those areas. For example, the LDL testing which for diabetics should occur annually, over the course of 2 years that will typically shoot up and meanwhile the value of the LDL, which you want to decrease, goes down. So those kinds of measures, we're seeing improvement."

"[I]mprovement in medication compliance."

"We also do the SF-12 and the scores vary somewhat due to the conditions as far as between mental health and the physical components. We'll sometimes see more improvement in the mental health component with some conditions that are typically chronic, i.e., congestive heart failure."

"[W]e also measure, where it's appropriate, days of . . . lost activity, and we'll see those drop pretty significantly over time."

"[We've seen a] decrease in inpatient admission and inpatient hospital days, an increase in pharmacy utilization, a decrease in [emergency room] usage . . . there are usually significant cost savings."

**Practice: MVP Healthcare, Schenectady, New York, and Williston, Vermont **

**Outcomes not published**

**Interview comment:**

"[Comparisons of] members who were in the program in the first half of 2002 to those who were not [show] that of people in the program, 90.4% had an LDL test within the year following their [hospital] discharge [versus] 80.1% of those not in the program."

## References

[1] Health, United States, 2006, with chartbook on trends in the health of Americans.

[2] (2006). Heart disease and stroke statistics — 2007 update.

[3] Stamler J, Stamler R, Neaton JD, Wentworth D, Daviglus ML, Garside D (1999). Low risk-factor profile and long-term cardiovascular and noncardiovascular mortality and life expectancy: findings for 5 large cohorts of young adult and middle-aged men and women. JAMA.

[4] Greenland P, Knoll MD, Stamler J, Neaton JD, Dyer AR, Garside DB (2003). Major risk factors as antecedents of fatal and nonfatal coronary heart disease events. JAMA.

[5] He J, Whelton PK (1999). Elevated systolic blood pressure and risk of cardiovascular and renal disease: overview of evidence from observational epidemiologic studies and randomized controlled trials. Am Heart J.

[6] Cohen JD (1997). A population-based approach to cholesterol control. Am J Med.

[7] (2000). Centers for Disease Control and Prevention. State-specific cholesterol screening trends—United States, 1991–1999. MMWR Morb Mortal Wkly Rep.

[8] Hajjar I, Kotchen TA (2003). Trends in prevalence, awareness, treatment, and control of hypertension in the United States, 1988–2000. JAMA.

[9] Ford ES, Mokdad AH, Giles WH, Mensah GA (2003). Serum total cholesterol concentrations and awareness, treatment, and control of hypercholesterolemia among U.S. adults: findings from the National Health and Nutrition Examination Survey, 1999 to 2000. Circulation.

[10] Healthy People 2010: heart disease and stroke.

[11] Rudd P, Miller NH, Kaufman J, Kraemer HC, Bandura A, Greenwald G (2004). Nurse management for hypertension. A systems approach. Am J Hypertens.

[12] Friedman RH, Kazis LE, Jette A, Smith MB, Stollerman J, Torgerson J (1996). A telecommunications system for monitoring and counseling patients with hypertension. Impact on medication adherence and blood pressure control. Am J Hypertens.

[13] McKenney JM, Munroe WP, Wright JT (1992). Impact of an electronic medication compliance aid on long-term blood pressure control. J Clin Pharmacol.

[14] Ornstein S, Jenkins RG, Nietert PJ, Feifer C, Roylance LF, Nemeth L (2004). A multimethod quality improvement intervention to improve preventive cardiovascular care: a cluster randomized trial. Ann Intern Med.

[15] Wagner EH, Austin BT, Von Korff M (1996). Organizing care for patients with chronic illness. Milbank Q.

[16] Matson Koffman DM, Goetzel RZ, Anwuri VV, Shore KK, Orenstein D, LaPier T (2005). Heart healthy and stroke free: successful business strategies to prevent cardiovascular disease. Am J Prev Med.

[17] Bodenheimer T, Wagner EH, Grumbach K (2002). Improving primary care for patients with chronic illness: the chronic care model, Part 2. JAMA.

[18] McLeroy KR, Bibeau D, Steckler A, Glanz K (1988). An ecological perspective on health promotion programs. Health Educ Q.

[19] (2001). Policy and environmental change: new directions for public health — final report.

[20] Chobanian AV, Bakris GL, Black HR, Cushman WC, Green LA, Izzo JL (2003). The seventh report of the Joint National Committee on Prevention, Detection, Evaluation, and Treatment of High Blood Pressure: the JNC 7 report. JAMA.

[21] Expert Panel on Detection, Evaluation, and Treatment of High Blood Cholesterol in Adults (2001). Executive summary of the third report of the National Cholesterol Education Program (NCEP) Expert Panel on Detection, Evaluation, and Treatment of High Blood Cholesterol in Adults (Adult Treatment Panel III). JAMA.

[22] Pearson TA, Blair SN, Daniels SR, Eckel RH, Fair JM, Fortmann SP (2002). AHA guidelines for primary prevention of cardiovascular disease and stroke: 2002 update: consensus panel guide to comprehensive risk reduction for adult patients without coronary or other atherosclerotic vascular diseases. American Heart Association Science Advisory and Coordinating Committee. Circulation.

[23] Smith SC, Blair SN, Bonow RO, Brass LM, Cerqueira MD, Dracup K (2001). AHA/ACC scientific statement: AHA/ACC guidelines for preventing heart attack and death in patients with atherosclerotic cardiovascular disease: 2001 update: a statement for healthcare professionals from the American Heart Association and the American College of Cardiology. Circulation.

[24] Healthy People 2010: access to quality health services.

[25] The chronic care model.

[26] Ockene JK, McBride PE, Sallis JF, Bonollo DP, Ockene IS (1997). Synthesis of lessons learned from cardiopulmonary preventive interventions in healthcare practice setting. Ann Epidemiol.

[27] Anwuri VV, Matson-Koffman DM Systems-level interventions for high blood pressure and high blood cholesterol control and prevention: a review of effective strategies in healthcare settings. Proceeding of the American Public Health Association 131st Annual Meeting and Exposition.

[28] (1996). U.S. Preventive Services Task Force. Guide to clinical preventive services.

[29] (2005). U.S. Preventive Services Task Force. Guide to clinical preventive services, 2001-2004.

[30] O'Connor PJ, Quiter ES, Rush WA, Wiest M, Meland JT, Ryu S (1999). Impact of hypertension guideline implementation on blood pressure control and drug use in primary care clinics. Jt Comm J Qual Improv.

[31] Borenstein JE, Graber G, Saltiel E, Wallace J, Ryu S, Archi J, comparative trial (2003). Physician-pharmacist comanagement of hypertension: a randomized, comparative trial. Pharmacotherapy. Pharmacotherapy.

[32] Stroebel RJ, Broers JK, Houle SK, Scott CG, Naessens JM (2000). Improving hypertension control: a team approach in a primary care setting. Jt Comm J Qual Improv.

[33] Bozovich M, Rubino CM, Edmunds J (2000). Effect of a clinical pharmacist-managed lipid clinic on achieving National Cholesterol Education Program low-density lipoprotein goals. Pharmacotherapy.

[34] Shaffer J, Wexler LF (1995). Reducing low-density lipoprotein cholesterol levels in an ambulatory care system. Results of a multidisciplinary collaborative practice lipid clinic compared with traditional physician-based care. Arch Intern Med.

[35] Vivian EM (2002). Improving blood pressure control in a pharmacist-managed hypertension clinic. Pharmacotherapy.

[36] Solomon DK, Portner TS, Bass GE, Gourley DR, Gourley GA, Holt JM (1998). Clinical and economic outcomes in the hypertension and COPD arms of a multicenter outcomes study. J Am Pharm Assoc (Wash).

[37] Bogden PE, Koontz LM, Williamson P, Abbott RD (1997). The physician and pharmacist team. An effective approach to cholesterol reduction. J Gen Intern Med.

[38] Ellis SL, Carter BL, Malone DC, Billups SJ, Okano GJ, Valuck RJ (2000). Clinical and economic impact of ambulatory care clinical pharmacists in management of dyslipidemia in older adults: the IMPROVE study. Impact of Managed Pharmaceutical Care on Resource Utilization and Outcomes in Veterans Affairs Medical Centers. Pharmacotherapy.

[39] Avanzini F, Corsetti A, Maglione T, Alli C, Colombo F, Torri V, shared guidelines (2002). Simple, shared guidelines raise the quality of antihypertensive treatment in routine care. Am Heart J.

[40] New JP, Mason JM, Freemantle N, Teasdale S, Wong LM, Bruce NJ (2003). Specialist nurse-led intervention to treat and control hypertension and hyperlipidemia in diabetes (SPLINT): a randomized controlled trial. Diabetes Care.

[41] Hill MN, Han HR, Dennison CR, Kim MT, Roary MC, Blumenthal RS (2003). Hypertension care and control in underserved urban African American men: behavioral and physiologic outcomes at 36 months. Am J Hypertens.

[42] Ditusa L, Luzier AB, Brady PG, Reinhardt RM, Snyder BD (2001). A pharmacy-based approach to cholesterol management. Am J Manag Care.

[43] Kiessling A, Henriksson P (2002). Efficacy of case method learning in general practice for secondary prevention in patients with coronary artery disease: randomised controlled study. BMJ.

[44] Goldstein MK, Lavori P, Coleman R, Advani A, Hoffman BB (2005). Improving adherence to guidelines for hypertension drug prescribing: cluster-randomized controlled trial of general versus patient-specific recommendations. Am J Manag Care.

[45] Moher M, Yudkin P, Wright L, Turner R, Fuller A, Schofield T (2001). Cluster randomised controlled trial to compare three methods of promoting secondary prevention of coronary heart disease in primary care. BMJ.

[46] Ho PM, Masoudi FA, Peterson ED, Grunwald GK, Sales AE, Hammermeister KE (2004). Cardiology management improves secondary prevention measures among patients with coronary artery disease. J Am Coll Cardiol.

[47] Asch SM, Baker DW, Keesey JW, Broder M, Schonlau M, Rosen M (2005). Does the collaborative model improve care for chronic heart failure?. Med Care.

[48] Becker DM, Yanek LR, Johnson WR, Garrett D, Moy TF, Reynolds SS (2005). Impact of a community-based multiple risk factor intervention on cardiovascular risk in black families with a history of premature coronary disease. Circulation.

[49] Bogden PE, Abbott RD, Williamson P, Onopa JK, Koontz LM (1998). Comparing standard care with a physician and pharmacist team approach for uncontrolled hypertension. J Gen Intern Med.

[50] Erickson SR, Slaughter R, Halapy H (1997). Pharmacists' ability to influence outcomes of hypertension therapy. Pharmacotherapy.

[51] Zillich AJ, Sutherland JM, Kumbera PA, Carter BL (2005). Hypertension outcomes through blood pressure monitoring and evaluation by pharmacists (HOME study). J Gen Intern Med.

[52] Denver EA, Barnard M, Woolfson RG, Earle KA (2003). Management of uncontrolled hypertension in a nurse-led clinic compared with conventional care for patients with type 2 diabetes. Diabetes Care.

[53] Okamoto MP, Nakahiro RK (2001). Pharmacoeconomic evaluation of a pharmacist-managed hypertension clinic. Pharmacotherapy.

[54] Brown AS, Cofer LA (2000). Lipid management in a private cardiology practice (the Midwest Heart experience). Am J Cardiol.

[55] Kinn JW, O'Toole MF, Rowley SM, Marek JC, Bufalino VJ, Brown AS (2001). Effectiveness of the electronic medical record in cholesterol management in patients with coronary artery disease (Virtual Lipid Clinic). Am J Cardiol.

[56] Kinn JW, Marek JC, O'Toole MF, Rowley SM, Bufalino VJ (2002). Effectiveness of the electronic medical record in improving the management of hypertension. J Clin Hypertens (Greenwich).

[57] Rogers MA, Small D, Buchan DA, Butch CA, Stewart CM, Krenzer BE (2001). Home monitoring service improves mean arterial pressure in patients with essential hypertension. A randomized, controlled trial. Ann Intern Med.

[58] Stamos TD, Shaltoni H, Girard SA, Parrillo JE, Calvin JE (2001). Effectiveness of chart prompts to improve physician compliance with the National Cholesterol Education Program guidelines. Am J Cardiol.

[59] Montgomery AA, Fahey T, Peters TJ, MacIntosh C, Sharp DJ (2000). Evaluation of computer based clinical decision support system and risk chart for management of hypertension in primary care: randomised controlled trial. BMJ.

[60] Bosworth HB, Olsen MK, Gentry P, Orr M, Dudley T, McCant F (2005). Nurse administered telephone intervention for blood pressure control: a patient-tailored multifactorial intervention. Patient Educ Couns.

[61] Bosworth H, Olsen K, Dudley T, Orr M, Goldstein M, Datta S (2008). Patient education and provider decision support to control blood pressure in primary care: a cluster randomized trial. Ann Intern Med.

[62] Artinian NT, Washington OG, Templin TN (2001). Effects of home telemonitoring and community-based monitoring on blood pressure control in urban African Americans: a pilot study. Heart Lung.

[63] Siskind A, Johnson M, Qureshi A, Solow B, Chesebro D, Oldham N (2000). The impact of automatic prescriptions on reducing low-density lipoprotein cholesterol levels. Eff Clin Pract.

[64] Khoury AT, Wan GJ, Niedermaier ON, LeBrun B, Stiebeling B, Roth M (2001). Improved cholesterol management in coronary heart disease patients enrolled in an HMO. J Healthc Qual.

[65] Stoddard AM, Palombo R, Troped PJ, Sorensen G, Will JC (2004). Cardiovascular disease risk reduction: the Massachusetts WISEWOMAN project. J Womens Health (Larchmt).

[66] Appel LJ, Champagne CM, Harsha DW, Cooper LS, Obarzanek E, Elmer PJ (2003). Effects of comprehensive lifestyle modification on blood pressure control: main results of the PREMIER clinical trial. JAMA.

[67] Vale MJ, Jelinek MV, Best JD, Dart AM, Grigg LE, Hare DL (2003). Coaching patients On Achieving Cardiovascular Health (COACH): a multicenter randomized trial in patients with coronary heart disease. Arch Intern Med.

[68] Headrick LA, Speroff T, Pelecanos HI, Cebul RD (1992). Efforts to improve compliance with the National Cholesterol Education Program guidelines. Results of a randomized controlled trial. Arch Intern Med.

[69] Kisioglu A, Aslan B, Ozturk M, Aykut M, Ilhan I (2004). Improving control of high blood pressure among middle-aged Turkish women of low socio-economic status through public health training. Croat Med J.

[70] Lin T, Chen CH, Chou P (2004). Impact of the high-risk and mass strategies on hypertension control and stroke mortality in primary health care. J Hum Hypertens.

[71] Rachmani R, Slavacheski I, Berla M, Frommer-Shapira R, Ravid M (2005). Treatment of high-risk patients with diabetes: motivation and teaching intervention: a randomized, prospective 8-year follow-up study. J Am Soc Nephrol.

[72] Davis AM, Vinci LM, Okwuosa TM, Huang ES (2007). Cardiovascular health disparities: a systematic review of health care interventions. Med Care Res Rev.

[73] Brinkerhoff RO (2003). The success case method: find out quickly what's working and what's not.

[74] IHI.org. Testing changes.

[75] Hypertension diagnosis and treatment (guideline).

[76] (1997). The sixth report of the Joint National Committee on prevention, detection, evaluation, and treatment of high blood pressure. Arch Intern Med.

[77] DMAA definition of disease management.

